# Postoperative hemorrhage in an elderly patient with a glioblastoma multiform and a calcified chronic subdural hematoma

**DOI:** 10.1186/1477-7819-12-110

**Published:** 2014-04-23

**Authors:** Jun Cai, Yanting Zhang, Xiaoxin Bai, Shaoxue Li, Jinhua Chen, Ruicong Chen, Hao Lin, Shengping Huang

**Affiliations:** 1 Department of Neurosurgery, Guangdong Provincial Hospital of Traditional Chinese Medicine (Affiliated Hospital at Guangzhou Higher Education Mega Center), 55 West Inner Circle Road, Guangzhou 510006, China; 2 The Second Institute of Clinical Medicine, Guangzhou University of Chinese Medicine, Guangzhou 510120, China; 3 Post-doctoral Research Center of Guangzhou University of Chinese Medicine, Guangzhou 510405, China

**Keywords:** Chronic subdural hematoma, Disseminated intravascular coagulation, Glioblastoma multiform

## Abstract

**Background:**

Cases with brain tumor and subdural hematoma are rare; surgical management of the elderly patients with a glioblastoma multiform (GBM) and a chronic subdural hematoma (CSDH) can be intractable.

**Case description:**

We report a 77-year-old patient, who had a left front lobe GBM and a giant, calcified, left frontoparietaloccipitotemporal CSDH. The patient recovered well from anesthesia after removal of the GBM and CSDH. However, the patient developed severe hemiplegia and aphasia because of the *in-situ* hemorrhage 1 day later. Laboratory tests indicated disseminated intravascular coagulation (DIC) leading to the postoperative hemorrhage. The patient was left with hemiparesis and alalia after the *in-situ* hematoma evacuation.

**Conclusions:**

We presume elderly patients have a higher incidence of postoperative hemorrhage in residual intracranial cavity owing to higher possibility to get DIC. A less aggressive surgical management could be a more appropriate choice.

## Background

Brain tumors and subdural hematomas are the most common intracranial lesions suffered by the elderly [1]. However, cases with both these two devastating maladies are infrequent. In addition, there are few data on the complications of management of elderly patients with brain tumors and chronic subdural hematomas. The surgical management of these cases can be problematic.

## Case presentation

**Figure 1:**
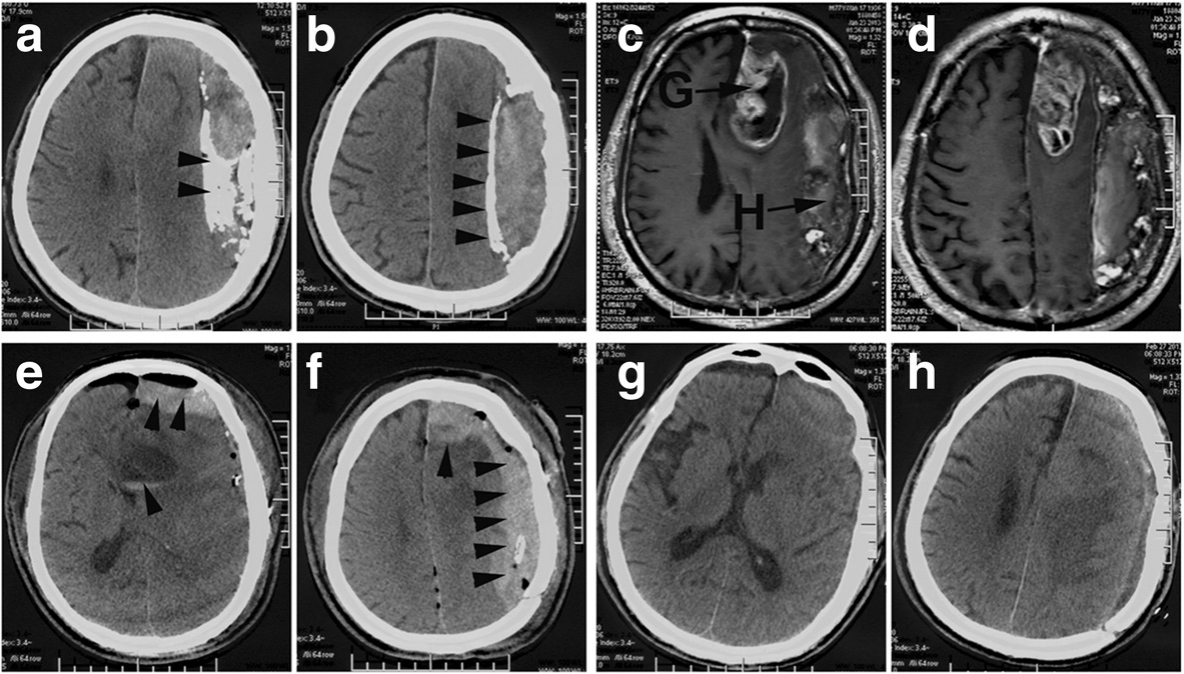
**The preoperative and postoperative images of CT and MRI. (a,b)** The CT images display the CSDH. The arrowheads point to the calcified membranes. **(c, d)** The MRI images show the GBM (G) and the CSDH (H). **(e, f)** The CT images indicate the *in-situ* hemorrhages after removal of the GBM and CSDH. **(g, h)**The postoperative CT images of the secondary craniotomy.

**Figure 2:**
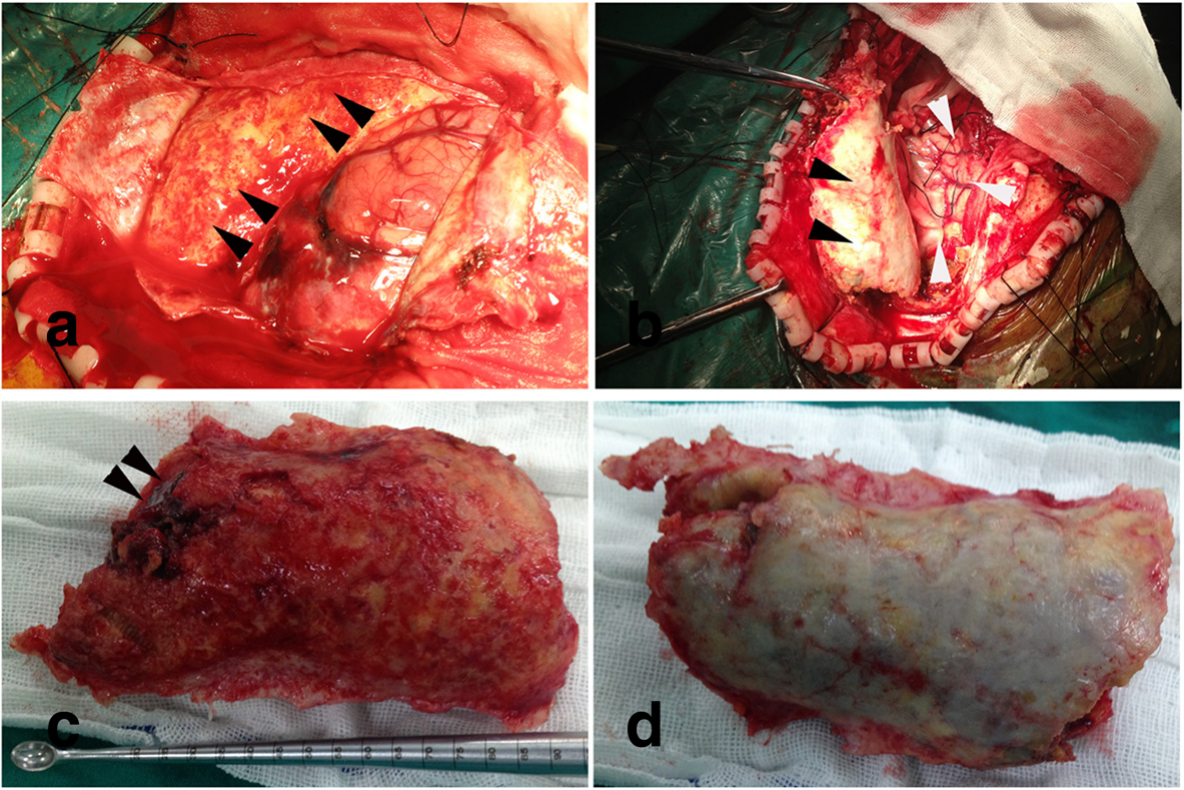
**The photographs of the CSDH. (a)** The panel displays the intact CSDH. **(b)** The panel indicates the isolated CSDH. **(c)** The panel shows the dural surface of the CSDH. Arrowheads point to the inner contents. **(d)** The panel shows the cortical surface of the CSDH.

A 77-year-old Chinese man was admitted to our hospital with a history of 2 weeks of hypomnesis and mental deterioration. Physical examination was unremarkable. He was diagnosed by computed tomography (CT) and magnetic resonance imaging (MRI) with a glioblastoma multiform (GBM) located in the left front lobe as well as a chronic subdural hematoma (CSDH) located in the left frontoparietaloccipitotemporal intracranial cavity (Figure 1a-d). A specific craniotomy was performed; the GBM was resected followed by evacuation of the giant and calcified CSDH (Figure 2a-d). The patient recoverexd from anesthesia well and no new neurological deficit was found after resuscitation. The next morning, the patient developed severe hemiplegia and aphasia due to the *in-situ* hemorrhage (Figure 1e-f). Postoperative laboratory work-up revealed there was a reduction of fibrinogen (0.69 g/L; normal: 2-4 g/L) and a huge increase of d-dimers (29,750 μg/L; normal: 0-500 μg/L), with platelet count, prothrombin time, and activated partial thromboplastin time all within normal limits. After correction of coagulopathy, secondary craniotomy was performed for the intracranial hematomas evacuation. The patient was left with hemiparesis and alalia after the second operation (Figure 1g-h) and discharged to take extensive neurological rehabilitation.

## Discussion

GBM is the most common primary malignant brain tumor in geriatric population [1]. Maximal surgical resection is one of the standard treatments for GBM [2]. Craniotomy remains the best option for evacuation of calcified CSDH [3]. Concerning this case, after GBM resection, we expanded the bone flap to thoroughly evacuate the calcified CSDH. No bleeding was found after the surgical procedures.

However, the *in-situ* hemorrhage was detected 1 day later. Postoperative laboratory analysis indicated the patient was in a state of prior disseminated intravascular coagulation (DIC) at that time. A diagnosis of DIC can be established if laboratory test results show a decreased fibrinogen and increased fibrin degradation products, such as d-dimers [4]. A number of cases are reported that DIC developed during or after removal of brain tumors [5-9], especially in the elderly. Development of DIC during or after craniotomy for brain tumor resection constitutes a very severe complication because nearly half of the patients in the reported cases died postoperatively [9].

There are four subtypes of GBM distinguished by gene expression patterns and clinical characteristics [10]. Studying GBMs using this new subtype classification therefore may accelerate our understanding of GBM pathology. A large sample set might help to determine whether the postoperative DIC correlate with the GBM subtypes, which can be used for optimal therapy.

## Conclusion

In summary, we presume elderly patients have a higher incidence of postoperative hemorrhage in residual intracranial cavity owing to higher possibility to get DIC. In the present case, a discreet two-step surgery which is comprised of resection of the GBM and evacuation of the giant and calcified CSDH could be more appropriate.

## Consent

Written informed consent was obtained from the patient and his daughter for publication of this case report and any accompanying images. A copy of the written consent is available for review by the Editor-in-Chief of this journal.

## Abbreviations

CSDH: Chronic subdural hematoma; CT: Computed tomography; DIC: Disseminated intravascular coagulation; GBM: Glioblastoma multiform; MRI: Magnetic resonance imaging.

## Competing interests

The authors declare that they have no competing interests.

## Authors’ contributions

JC took a part in the surgical and medical management of the patient, and drafted the manuscript. YZ took a part in the medical management of the case and assisted in preparation of the manuscript. XB took a part in the first craniotomy and revised the manuscript. SL and JC participated in the second operation. RC and HL participated in the rehabilitation of the patient. SH supervised the whole management of the patient and took a part in the second craniotomy. All authors read and approved the final manuscript.

## Authors’ information

JC, XB, SL, JC, and SH are neurosurgeons. YZ, who specializes in intensive care therapy, is a neurologist. RC and HL are physical therapists.
